# Conditional Deletion of *Foxg1* Delayed Myelination during Early Postnatal Brain Development

**DOI:** 10.3390/ijms241813921

**Published:** 2023-09-10

**Authors:** Guangliang Cao, Congli Sun, Hualin Shen, Dewei Qu, Chuanlu Shen, Haiqin Lu

**Affiliations:** 1 Department of Human Anatomy, School of Medicine, Southeast University, Nanjing 210009, China; 101011390@seu.edu.cn (G.C.); 220203900@seu.edu.cn (H.S.); 101012391@seu.edu.cn (D.Q.); 2 Department of Physiology, School of Medicine, Southeast University, Nanjing 210009, China; 230208416@seu.edu.cn; 3 Department of Pathophysiology, School of Medicine, Southeast University, Nanjing 210009, China; 101010570@seu.edu.cn

**Keywords:** *Foxg1*, delayed myelination, PDGFRα, oligodendrocyte precursor cells, cell cycle

## Abstract

*FOXG1* (forkhead box G1) syndrome is a neurodevelopmental disorder caused by variants in the *Foxg1* gene that affect brain structure and function. Individuals affected by *FOXG1* syndrome frequently exhibit delayed myelination in neuroimaging studies, which may impair the rapid conduction of nerve impulses. To date, the specific effects of FOXG1 on oligodendrocyte lineage progression and myelination during early postnatal development remain unclear. Here, we investigated the effects of *Foxg1* deficiency on myelin development in the mouse brain by conditional deletion of *Foxg1* in neural progenitors using *NestinCreER;Foxg1^fl/fl^* mice and tamoxifen induction at postnatal day 0 (P0). We found that *Foxg1* deficiency resulted in a transient delay in myelination, evidenced by decreased myelin formation within the first two weeks after birth, but ultimately recovered to the control levels by P30. We also found that *Foxg1* deletion prevented the timely attenuation of platelet-derived growth factor receptor alpha (PDGFRα) signaling and reduced the cell cycle exit of oligodendrocyte precursor cells (OPCs), leading to their excessive proliferation and delayed maturation. Additionally, *Foxg1* deletion increased the expression of Hes5, a myelin formation inhibitor, as well as Olig2 and Sox10, two promoters of OPC differentiation. Our results reveal the important role of *Foxg1* in myelin development and provide new clues for further exploring the pathological mechanisms of *FOXG1* syndrome.

## 1. Introduction

Myelin is a lipid-rich sheath that envelops the axons of neurons and facilitates the rapid conduction of nerve impulses [1]. Oligodendrocytes (OLs) are the myelin-forming cells in the central nervous system (CNS). They originate from neural progenitor cells (NPCs) of the ventricular zones, which first differentiate into oligodendrocyte precursor cells (OPCs) expressing specific antigens such as platelet-derived growth factor receptor alpha (PDGFRα) and neuron-glial antigen 2 (NG2). OPCs then migrate from the ventricular zones to various regions of the developing CNS, where they undergo terminal differentiation into myelinating OLs. Once committed to the OLs fate, OPCs exit the cell cycle and express myelin-associated proteins such as proteolipid protein 1 (PLP1) and myelin basic protein (MBP). Finally, OLs wrap their extensions around the nerve fibers, forming myelin sheaths that match the size and activity of the nerves [2,3]. The timing and extent of myelination are influenced by multiple intrinsic and extrinsic factors, such as transcription factors, growth factors, and neuronal activity [4,5]. Aberrant myelination during development is the primary manifestation of white matter injury in infancy and early childhood and is closely linked to a variety of neurodevelopmental disorders [6,7].

FOXG1, a member of the forkhead-box family of transcription factors, is essential for orchestrating the intricate molecular events that govern proper brain development and organization, ranging from telencephalic pattern formation [8,9], NPCs proliferation and differentiation [10], neuronal migration and specialization [11,12,13,14], to the establishment of functional cortical circuitry [15]. In the clinic, pathogenic variants in *FOXG1* cause a severe neurodevelopmental disorder known as *FOXG1* syndrome, which is characterized by intellectual disability, global developmental delay, microcephaly, dyskinesia, epilepsy, and autistic features [16]. In addition, multiple studies have reported evidence of delayed or impaired myelination in *FOXG1* syndrome patients, as shown by reduced white matter volume and altered diffusion tensor imaging parameters [17,18]. Intriguingly, a recent study has shown that *Foxg1* deletion in NPCs enhances OL differentiation and remyelination in adult demyelinated mice rather than leading to delays or impairments in myelination [19]. This suggests that *Foxg1* might have a different role in myelin formation during early development and adulthood. However, no reports have been found to address how FOXG1 influences myelination in early development.

In the current study, by crossing *NestinCreER* mice with *Foxg1^fl/fl^* mice, we selectively deleted *Foxg1* in NPCs upon tamoxifen (TM) induction at P0. This allows us to study the role of *Foxg1* in oligodendrogenesis and myelin formation during brain development. We found that during the first two weeks after birth, *Foxg1* deficiency led to a decrease in the number of mature OLs, resulting in impaired myelination. In contrast, there was an increase in the number of OLIG2+ total OL lineage cells and PDGFRα+ OPCs during this period. However, this effect was transient and recovered by P30. We have also identified potential molecular mechanisms underlying the delayed myelination, including hindered attenuation of the PDGFRα signal, reduced OPC cell cycle exit, and increased expression of Hes5, Olig2, and Sox10. This study provides new insights into the role of *Foxg1* in timely myelination during the early postnatal stages, which may deepen our understanding of the pathogenesis of *FOXG1* syndrome and other myelin-related disorders.

## 2. Results

### 2.1. Foxg1 Deletion Leads to Defect in Myelination at Early Postnatal Period

**Figure 1:**
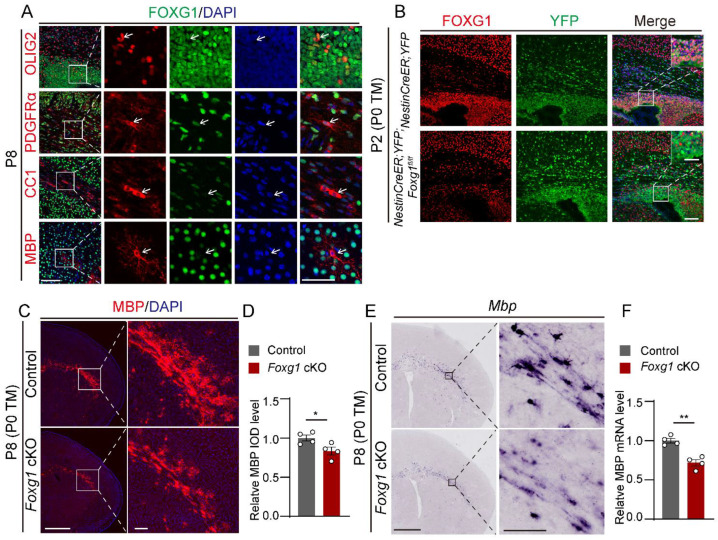
Defects in myelin development in newborn mice with FOXG1 deletion. (**A**) Immunofluorescence co-staining of FOXG1 (green) and OL lineage markers OLIG2, PDGFRα, CC1, and MBP (red) in the forebrain of P8 mice. Nuclei are counterstained with DAPI (blue). The white arrows point to different OL lineage cells. Scale bar, 100 μm. White boxes indicate regions shown at higher magnification on the right. Scale bar, 50 μm. (**B**) Immunofluorescence co-staining of FOXG1 (red) and YFP (green) in the forebrain of P2 *NestinCreER;Foxg1^fl/fl^;YFP* mice, showing efficient deletion of *Foxg1* in YFP-labeled progenitors. Scale bar, 100 μm. White boxes indicate regions shown at higher magnification at the top right. Scale bar, 50 μm. (**C**) Immunofluorescence staining for MBP (red) in the forebrain of P8 mice showed reduced MBP expression in the lateral external capsule of *Foxg1* cKO mice compared to control mice. Scale bar, 1 mm. Black boxes indicate regions shown at higher magnification at right. Scale bar,100 μm. (**D**) Quantification of MBP immunostaining intensity by integrated optical density (IOD) in the lateral external capsule of P8 mice. (**E**) In situ hybridization for MBP mRNA expression in the forebrain of P8 mice, showing decreased MBP mRNA levels in *Foxg1* cKO mice compared to control mice. Scale bar, 1 mm. Black boxes indicate regions shown at higher magnification at right. Scale bar, 100 μm. (**F**) Relative quantification of MBP mRNA expression by RT-qPCR in the forebrain of P8 mice. Values are mean ± SEM. * *p* < 0.05, ** *p* < 0.01 by Student’s *t*-test, compared to the control group.

Previous studies have shown that FOXG1 is highly expressed in NPCs [20]. Here, we first examined its expression profile in OL lineage cells. As shown in Figure 1A, the expression of FOXG1 was observed in OLIG2^+^ and PDGFRα^+^ OPCs in P8 wild-type mice. In contrast, FOXG1 was downregulated rapidly and was almost undetectable in CC1^+^ and MBP^+^ myelinating OLs, indicating its significant contribution to the initial stages of OL differentiation. Then, by crossing *Foxg1^fl/fl^* mice with *Nestin-creER* mice and employing TM induction at P0, we studied the effect of *Foxg1* deletion on the entire OL lineage development, including the determination of cell fate from NPCs to OPCs, as well as subsequent differentiation and maturation processes. To verify the efficiency of Foxg1 ablation, we introduced the ROSA-YFP reporter line to track *Foxg1*-deficient cells. Using double immunostaining for YFP and FOXG1, we observed strong co-localization of FOXG1 staining with YFP in *NestinCreER;YFP* mice at P2. In contrast, FOXG1 was undetectable in YFP^+^ progenitor cells of *NestinCreER;YFP;Foxg1^fl/fl^* mice, indicating successful ablation (Figure 1B).

To investigate the effect of *Foxg1* deletion on early postnatal myelination, we first performed MBP immunostaining in the forebrain of mice at P8, a stage when myelination becomes visible. As expected, strong immunoreactivity for MBP was observed in the external capsule of control mice at P8. In contrast, the intensity of MBP immunostaining was significantly reduced in the corresponding brain regions of *NestinCreER;Foxg1^fl/fl^* (hereafter referred as *Foxg1* cKO) mice, as shown by a diminished band of MBP-positive staining (Figure 1C,D). Then, in situ hybridization using probes for MBP mRNA also revealed a significant decrease in MBP mRNA signals in *Foxg1* cKO mice at P8, indicating impaired myelination (Figure 1E). Finally, RT-qPCR analysis further confirmed these findings (Figure 1F). In conclusion, our results indicate that the deletion of *Foxg1* disrupts myelin development in the early postnatal period.

### 2.2. Transient Defects in Myelination Are Observed in Foxg1 cKO Mice

**Figure 2:**
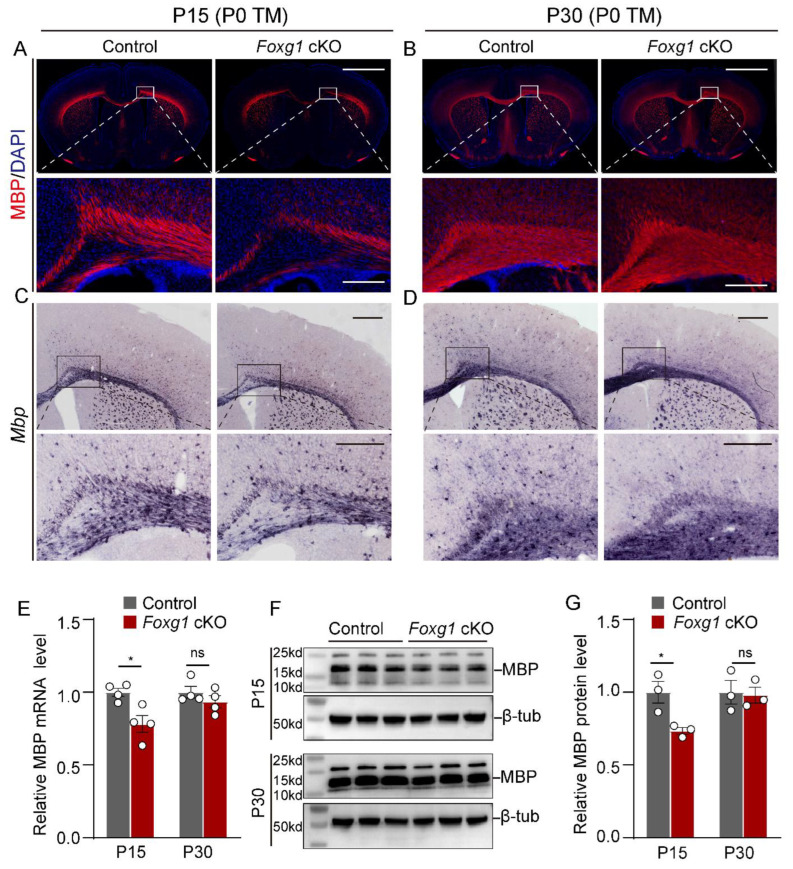
*Foxg1* cKO mice exhibit transient defects in myelination. (**A**,**B**) Immunofluorescence staining of MBP (red) and DAPI (blue) in the forebrain sections of control and *Foxg1* cKO mice at P15 (**A**) and P30 (**B**). Scale bar, 2 mm. The white boxes indicate regions shown at higher magnification. Scale bar, 200 μm. (**C**,**D**) In situ hybridization of MBP mRNA (purple) in the forebrain sections of control and *Foxg1* cKO mice at P15 (**C**) and P30 (**D**). Scale bar, 500 μm. The black boxes indicate regions shown at higher magnification. Scale bar, 200 μm. (**E**) RT-qPCR analysis of MBP mRNA levels in brain lysates from control and *Foxg1* cKO mice at P15 and P30. Data are normalized to GAPDH and expressed as fold change relative to control. *n* = 4 per group. (**F**) Western blot analysis of MBP protein levels in the brain lysates of control and *Foxg1* cKO mice at P15 and P30. β-Tubulin was used as a loading control. *n* = 3 per group. (**G**) Densitometric quantification of MBP protein levels. Data are normalized to β-tubulin and expressed as fold change relative to control. Error bars represent SEM. ns, not significant, * *p* < 0.05 by Student’s *t*-test.

To determine whether the myelination defect in *Foxg1* cKO mice at P8 was transient or persistent, we detected myelin expression patterns with MBP by immunostaining and in situ hybridization at P15 and P30. Compared with control mice, we found that the fluorescence and hybridization signal for Mbp decreased obviously in *Foxg1* cKO mice at P15, especially in the corpus callosum (CC) and cortex (Figure 2A,C). However, this decrease was not observed at P30 (Figure 2B,D). Moreover, we performed RT-qPCR (Figure 2E) and Western blot analysis (Figure 2F,G) to quantify the level of MBP expression in forebrain lysates from both control and *Foxg1* cKO mice at P15 and P30. The results obtained from both methods confirmed the reduced MBP expression in *Foxg1* cKO mice at P15. However, at P30, there was no significant difference in MBP levels between the two groups. These results indicate that *Foxg1* deletion leads to a transient delay, rather than a permanent block, of myelination.

### 2.3. Foxg1 Deletion Lead to Impaired OLs Maturation

**Figure 3:**
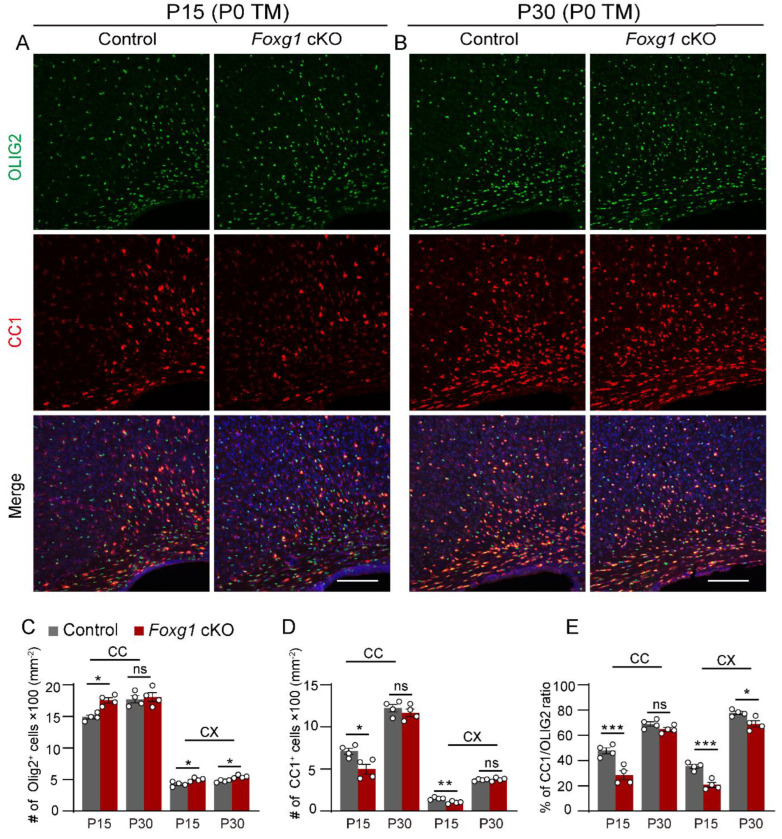
*Foxg1* deletion leads to impaired OL maturation. (**A**,**B**) Immunofluorescence staining of CC1 (red) and OLIG2 (green) in the forebrain of control and *Foxg1* cKO mice at P15 (**A**) and P30 (**B**). DAPI (blue) was used to label nuclei. Scale bar, 100 μm. (**C–E**) Quantification of OLIG2^+^ cells (**C**), CC1^+^ cells (**D**), and CC1/OLIG2 ratio (**E**) in the CC and cortex of control and *Foxg1* cKO mice at P15 and P30. In each subregion, *n* = 4 per group. Error bars represent SEM. ns, not significant, * *p* < 0.05, ** *p* < 0.01, *** *p* < 0.001 by one-way ANOVA followed by Bonferroni post hoc test in each subregion. CC, corpus callosum; CX, cortex.

To determine whether the delay in myelination observed in *Foxg1* cKO mice was due to impaired OL maturation, we measured the number of total OL lineage cells (OLIG2^+^ cells) and mature myelinating OLs (CC1^+^ cells) in the CC and cortex at P15 and P30. At P15, we observed an increase in the density of OLIG2^+^ cells in both brain regions of *Foxg1* cKO mice compared to control mice (Figure 3A,C; CC: increased by 17.7%, *p* < 0.05; cortex: increased by 17.5%, *p* < 0.05). In contrast, the density of CC1^+^ cells showed a significant decrease (Figure 3A,D; CC: decreased by 30.1%, *p* < 0.05; cortex: decreased by 31.3%, *p* < 0.01). By P30, the density of OLIG2^+^ cells in the CC of *Foxg1* cKO mice returned to levels comparable to those of control mice (*p* > 0.05) (Figure 3B,C). However, in the cortex, the density of OLIG2^+^ cells remained elevated, showing an increase of over 14% (*p* < 0.05) compared with the control mice (Figure 3B,C). No significant differences were observed in the density of CC1^+^ cells between *Foxg1* cKO and control mice in either region at P30 (Figure 3B,D). 

We then assessed the maturity status of OLs by calculating the CC1/OLIG2 ratio. At P15, both the CC and cortex of *Foxg1* cKO mice showed a significant decrease in the CC1/OLIG2 ratio (Figure 3E; CC: decreased by 40.4%, *p* < 0.001; cortex: decreased by 41.4%, *p* < 0.001). However, by P30, the CC1/OLIG2 ratio in the CC of *Foxg1* cKO mice had recovered to levels comparable to controls (*p* > 0.05), while in the cortex, it remained lower than controls (decreased by 11.2%, *p* < 0.05) (Figure 3E). Overall, these findings suggest that the deletion of *Foxg1* results in a delay in OL maturation.

**Figure 4:**
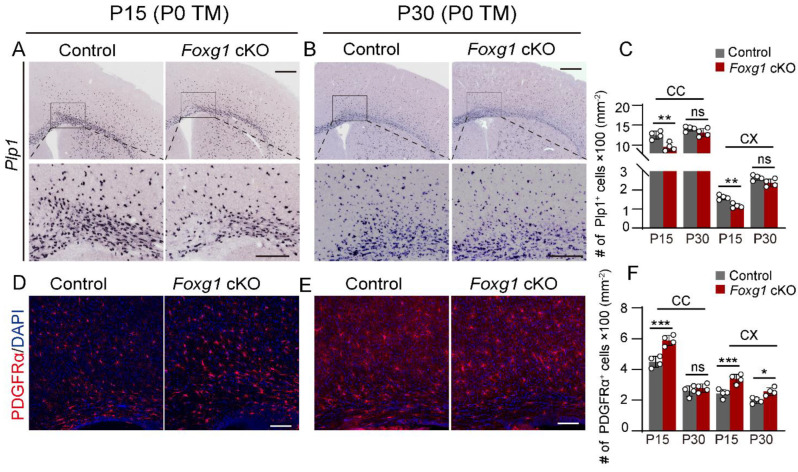
*Foxg1* deletion leads to an increased number of PDGFRα^+^ OPCs. (**A**,**B**) In situ hybridization of Plp1 mRNA (purple) in the CC and cortex of control and *Foxg1* cKO mice at P15 (**A**) and P30 (**B**). Scale bar, 500 μm. The black boxes indicate regions shown at higher magnification. Scale bar, 200 μm. (**C**) Quantification of Plp1^+^ cells in the CC and cortex of control and *Foxg1* cKO mice at P15 and P30. *n* = 4 per group. (**D**,**E**) Immunofluorescence staining of PDGFRα (red) in the forebrain of control and *Foxg1* cKO mice at P15 (**D**) and P30 (**E**). DAPI (blue) was used to label nuclei. Scale bar, 100 μm. (**F**) Quantification of PDGFRα^+^ cells in the CC and cortex of control and *Foxg1* cKO mice at P15 and P30. *n* = 4 per group. Error bars represent SEM. ns, not significant, * *p* < 0.05, ** *p* < 0.01, *** *p* < 0.001 by one-way ANOVA followed by Bonferroni post hoc test in each subregion. CC, corpus callosum; CX, cortex.

To further confirm the delayed maturation of OLs, in situ hybridization of Plp1 was performed. Consistently, a significant reduction in the density of Plp1^+^ cells was observed in both the CC and cortex in *Foxg1* cKO mice at P15 (Figure 4A,C; CC: decreased by 23.5%, *p* < 0.01; cortex: decreased by 28%, *p* < 0.01). In contrast, by P30, there was a significant recovery in the density of Plp1^+^ cells in both regions, and no significant differences were detected between the two groups of mice (Figure 4B,C). These results provide additional evidence for the delayed maturation of OLs in *Foxg1* cKO mice.

We speculated that the reduced mature myelinating OLs could result from the impaired terminal differentiation of OPC. To test this speculation, we conducted PDGFRα immunostaining on the forebrains to investigate the potential elevation of OPC numbers caused by delayed differentiation. As expected, there was an increase in the density of PDGFRα^+^ cells in both the CC and cortex of *Foxg1* cKO mice compared to control mice at P15 (Figure 4D,F, CC: increased by 31.0%, *p* < 0.001; cortex: increased by 39.5%, *p* < 0.01). By P30, the density of PDGFRα^+^ cells in the cortex remained elevated in *Foxg1* cKO mice with an increase of 30.2% (*p* < 0.05), while the density in the CC was comparable to that of control mice (Figure 4E,F).

In addition, we conducted immunostaining using OLIG2 and CASPASE-3 to investigate whether apoptosis contributed to the impairment of myelination. However, no significant alterations were observed in both groups of mice at P8 and P15. Taken together, these results suggest that *Foxg1* is an important regulator of OL maturation, and its loss causes transient but not permanent defects in the maturation process.

### 2.4. Foxg1 Deletion Induces Cell−Autonomous Impairment in OLs Maturation

**Figure 5:**
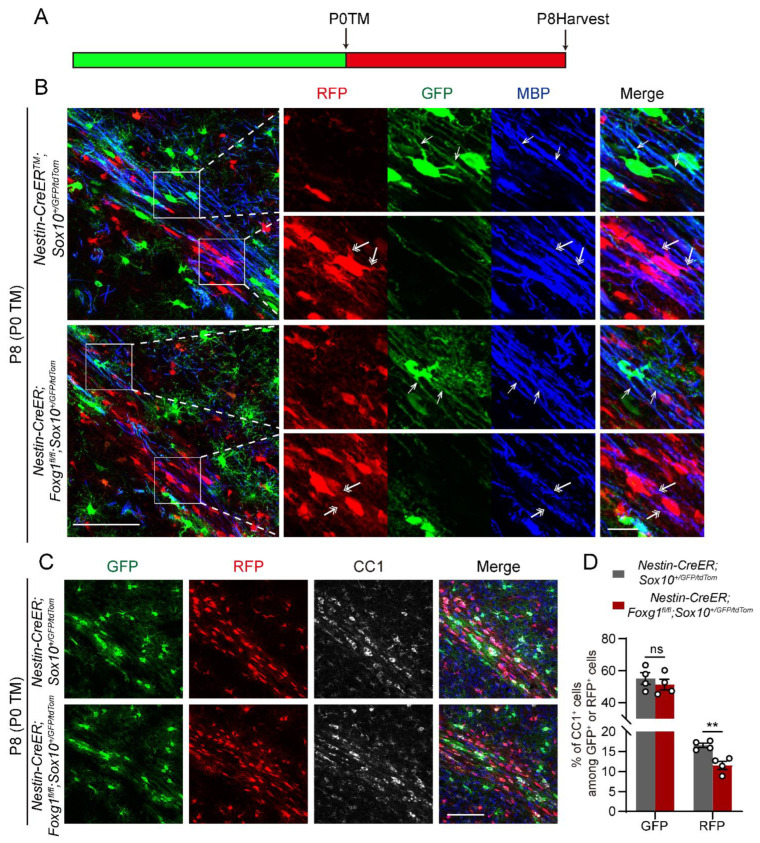
Cell-autonomous impairment of OL maturation caused by *Foxg1* deletion. (**A**) Schematic of the experimental design. *Nestin-Cre;Foxg1^fl/fl^;Sox10^GFP/tdTOM^* triple-transgenic mice were treated with TM at P0 to induce *Foxg1* deletion. At P8, the brains were harvested and analyzed for OL maturation. (**B**) Immunofluorescence staining of GFP (green), RFP (red), and MBP (blue) in the forebrain of control and *Foxg1* cKO mice at P8. Scale bar, 100 μm. The white boxes indicate regions shown at higher magnification. Single arrowheads point to the myelin segments expressed by non-recombined cells, while double arrowheads point to the myelin segments expressed by recombined cells in both groups of mice. Scale bar, 20 μm. (**C**) Immunofluorescence staining of GFP (green), RFP (red), and CC1 (grey) in control and *Foxg1* cKO mice at P8. Scale bar, 100 μm. (**D**) Quantification of the percentage of CC1^+^ cells among RFP^+^ and GFP^+^ cells in the external capsule of control and *Foxg1* cKO mice at P8. *n* = 4 per group. Error bars represent SEM. ns, not significant, ** *p* < 0.01 by Student’s *t*-test.

NPCs in the early postnatal subventricular zone (SVZ) retain high pluripotency, being able to generate not only OLs but also other types of cells, such as astrocytes and neurons. Previous studies have found that *Foxg1* deficiency leads to an increase in astrocytes [21,22], which may also have an indirect effect on myelin development in the current study. Therefore, to investigate whether the myelination defect in *Foxg1* cKO mice was mainly due to cell−autonomous effects or changes in the extracellular environment, we generated *NestinCreER;Foxg1^fl/fl^;Sox10^GFP/tdTOM^* triple−transgenic mice. In these mice, all Sox10^+^ OL lineage cells that ever express inducible Cre under the control of the Nestin promoter are permanently labeled with tomato red, while those that never express Cre remain GFP−labeled. Here, the control group consisted of *NestinCreER;Sox10^GFP/tdTOM^* mice. TM induction was conducted at P0, and tissue analysis was performed at P8 (Figure 5A).

To investigate the myelin formation of recombined and non−recombined oligodendrocytes in both control and experimental groups of mice, we performed triple immunofluorescence staining for GFP, RFP, and MBP. We observed that in *NestinCreER;Foxg1^fl/fl^;Sox10^GFP/tdTOM^* mice, the RFP^+^ recombined cells exhibited slightly smaller cell bodies, shorter processes, fewer branches, and sparser myelin segments compared to the *NestinCreER;Sox10^GFP/tdTOM^* mice. Conversely, there was no discernible distinction observed between the GFP^+^ non-recombined cells in the two groups (Figure 5B).

To evaluate the maturation status of recombined and non-recombined OLs in control and experimental mice, we conducted triple immunofluorescence staining for GFP, RFP, and CC1. In *NestinCreER;Sox10^GFP/tdTOM^* mice, the percentage of CC1^+^ cells among RFP^+^ cells was 16.6 ± 0.6%. However, in *NestinCreER;Foxg1^fl/fl^;Sox10^GFP/tdTOM^* mice, this percentage significantly decreased to 11.6 ± 1.0% (*p* < 0.01), indicating impaired OL maturation. Moreover, the proportion of CC1^+^ mature OLs among GFP^+^ cells, which primarily originated before TM induction, showed no significant difference between the two groups. The values were 55.1 ± 3.6% and 51.3 ± 3.3% (*p* > 0.05) for *NestinCreER;Sox10^GFP/tdTOM^* and *NestinCreER;Foxg1^fl/fl^;Sox10^GFP/tdTOM^* mice, respectively (Figure 5C,D). These findings suggest that *Foxg1* deletion impairs the maturation of OL lineage cells, mainly in a cell−autonomous manner.

### 2.5. Foxg1 Promotes Cell Cycle Exit in OPCs while Maintaining Proliferation in NPCs 

**Figure 6:**
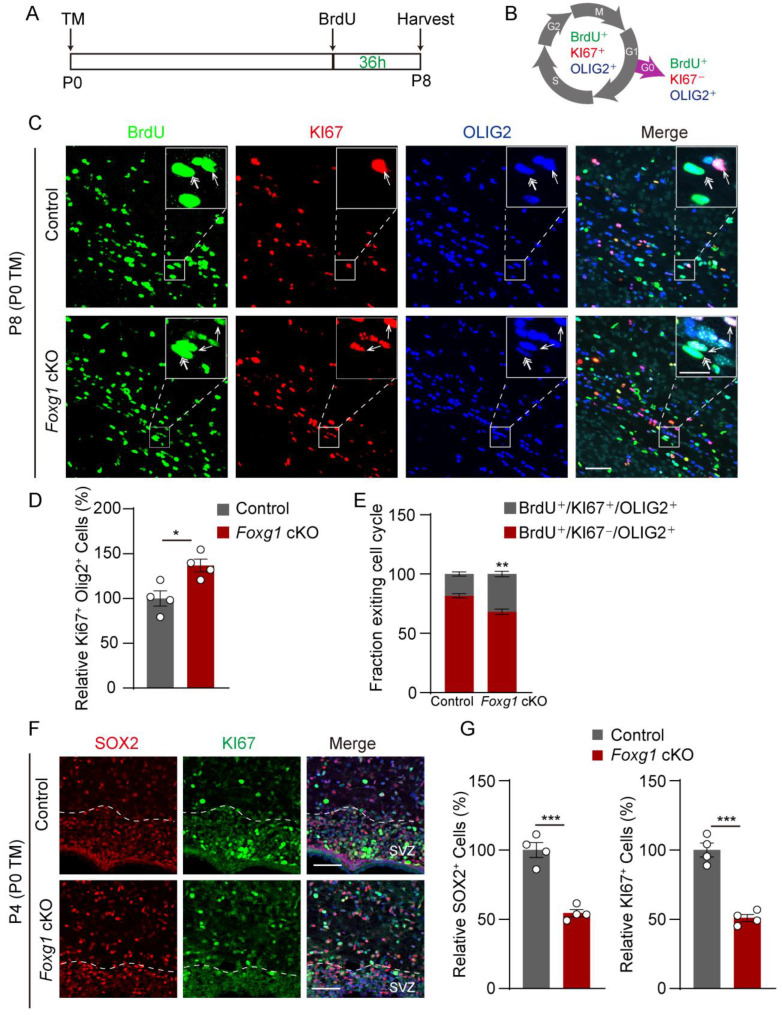
*Foxg1* maintains proliferation in NPCs while promoting cell cycle exit in OPCs. (**A**) Schematic diagram of the BrdU incorporation assay. (**B**) Schematic diagram of the cell cycle exit of OPCs, showing OPCs in the cell cycle (BrdU^+^/KI67^+^/OLIG2^+^) and those that had exited the cell cycle (BrdU^+^/KI67^−^/OLIG2^+^). (**C**) Immunofluorescence staining of BrdU (green), KI67 (red), and OLIG2 (blue) in the CC of control and *Foxg1* cKO mice. Scale bar, 20 μm. White boxes indicate regions shown at higher magnification in the insets. Single arrowheads indicate BrdU^+^/KI67^+^/OLIG2^+^ cells, while double arrowheads indicate BrdU^+^/KI67^−^/OLIG2^+^ cells in both groups of mice. Scale bar, 200 μm. (**D**) Quantification of the total number of KI67^+^/OLIG2^+^ cells in the CC of control and *Foxg1* cKO mice. (**E**) Quantification of the cell cycle exit index of OPCs in the external capsule of control and *Foxg1* cKO mice. The cell cycle exit index was calculated as the percentage of KI67^-^ cells among all BrdU^+^/OLIG2^+^ cells. (**F**) Immunofluorescence staining of SOX2 (red) and KI67 (green) in the SVZ region of control and *Foxg1* cKO mice at P4. DAPI (blue) was used to label nuclei. Scale bar, 50 μm. (**G**) Quantification of the total number of SOX2^+^ and KI67^+^ cells in the SVZ region of control and *Foxg1* cKO mice at P4. *n* = 4 per group. Error bars represent SEM. * *p* < 0.05, ** *p* < 0.01, *** *p* < 0.001 by Student’s *t*-test.

As withdrawal from the cell cycle is a crucial checkpoint for OPC differentiation into myelinating cells, we hypothesized that *Foxg1* deletion may affect this process. To test this hypothesis, we performed immunostaining for BrdU, KI67, and OLIG2 on coronal sections of the forebrain of P8 mice that had received BrdU injections 36 h earlier, when OPCs proliferation and differentiation were active in the white matter (Figure 6A). By co-staining for these markers, OPCs in the cell cycle were identified as BrdU^+^/KI67^+^/OLIG2^+^, while those that had exited the cell cycle were identified as BrdU^+^/KI67^−^/OLIG2^+^ (Figure 6B). The density of KI67^+^/OLIG2^+^ cells was significantly increased in *Foxg1* cKO mice compared to control mice, indicating enhanced proliferation of OPCs (Figure 6C,D). Additionally, the proportion of BrdU^+^/KI67^−^/OLIG2^+^ cells among all BrdU^+^/OLIG2^+^ cells was significantly lower in *Foxg1* cKO mice compared to control mice (Figure 6C,E; control: 81.6 ± 1.7% vs. *Foxg1* cKO: 68.8 ± 2.3%, *p* < 0.01), suggesting impaired cell cycle exit of OPCs. 

Based on these results, we further investigated the impact of *Foxg1* deletion on NPC proliferation. In the brains of P4 mice, we conducted immunostaining for SOX2 and KI67 and found a significant reduction in the number of SOX2^+^ and KI67^+^ cells in the SVZ of *Foxg1* cKO mice (Figure 6F,G). This result is consistent with previous studies suggesting that *Foxg1* plays a crucial role in maintaining stem cell proliferation and preventing premature differentiation.

Taken together, the above findings indicate that *Foxg1* may have a dual role in the cell cycle regulation of OPCs and NPCs, as it is involved in promoting OPCs cell cycle exit and maintaining NPCs proliferation.

### 2.6. Foxg1 Deletion Leads to Imbalance of the Regulatory Mechanisms of Myelination

**Figure 7:**
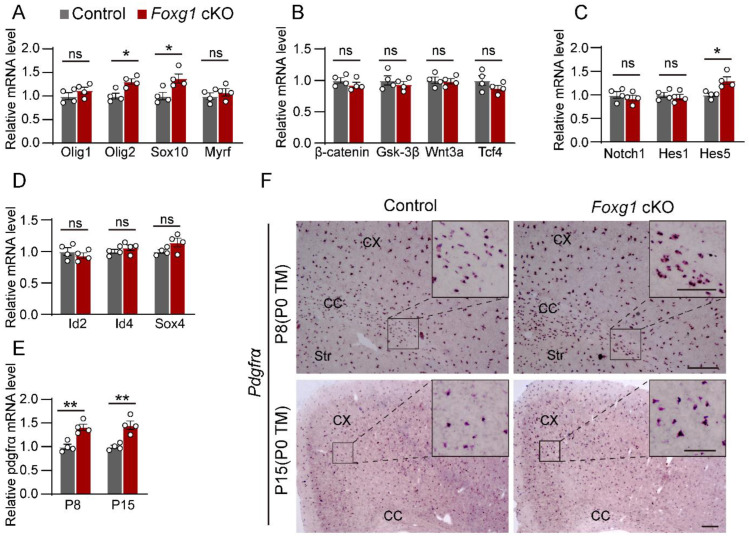
*Foxg1* deletion leads to an imbalance of the regulatory mechanisms of myelination. (**A**–**D**) RT-qPCR analysis of the expression levels of genes involved in OL differentiation and myelination in control and *Foxg1* cKO mice at P8, including: (**A**) genes that promote OL differentiation and myelination (Olig1, Olig2, Sox10, and Myrf), (**B**) genes that are components of the Notch signaling pathway (Notch1, Hes1, and Hes5), (**C**) genes that are components of the Wnt signaling pathway (Gsk3β, β-catenin, Wnt3a, and Tcf4), and (**D**) genes that inhibit myelination (Sox4, ID2, and ID4). (**E**) RT-qPCR analysis of the expression levels of Pdgfrα in control and *Foxg1* cKO mice at P8 and P15. Data are normalized to GAPDH and expressed as fold change relative to control. *n* = 4 per group. Error bars represent SEM. ns, not significant, * *p* < 0.05, ** *p* < 0.01, by Student’s *t*-test. (**F**) In situ hybridization of Pdgfrα mRNA (purple) in the forebrain sections of control and *Foxg1* cKO mice at P8 and P15. Scale bar, 200 μm. Black boxes indicate regions shown at higher magnification in the insets. Scale bar, 100 μm. CX, cortex; CC, corpus callosum; Str, striatum.

To elucidate the underlying molecular mechanisms, we performed RT-qPCR to measure the effect of *Foxg1* deletion on genes related to myelin formation. We found that *Foxg1* deletion increased the expression of Olig2 and Sox10, which are transcription factors that positively regulate OL lineage progression at P8 (Figure 7A; Olig2: increased by 31.0%, *p* < 0.05; Sox10: increased by 37.4%, *p* < 0.05). However, Olig1 and Myrf levels did not change significantly at this time point (Figure 7A).

We also examined some signaling pathway components closely associated with myelination, including Notch1, Hes1, and Hes5 from the Notch signaling pathway [23] (Figure 7B), as well as Gsk3β, β-catenin, Wnt3a, and Tcf4 from the Wnt signaling pathway [24] (Figure 7C). Additionally, we investigated several factors known to inhibit myelination, such as Sox4 [25], Id2, Id4 [26] (Figure 7D), and Pdgfrα (Figure 7E). Most of these factors were unaffected by *Foxg1* deletion at P8, except for Hes5 and Pdgfrα, which were significantly elevated in *Foxg1* cKO mice (Figure 7B,E; Hes5: increased by 30.5%, *p* < 0.05; Pdgfra: increased by 41.2%, *p* < 0.01). At P15, the expression level of Pdgfrα in *Foxg1* cKO mice was still more than 45.7% (*p* < 0.01) higher than that in the control mice (Figure 7E). To further confirm the pronounced upregulation of Pdgfrα expression, we performed in situ hybridization. The Pdgfrα staining intensity in *Foxg1* cKO mice was markedly higher than that in the control group at both P8 and P15 (Figure 7F). These results suggest that *Foxg1* is a multifunctional regulator that modulates both positive and negative factors involved in myelination, ultimately determining the timing and extent of myelin formation.

## 3. Discussion

Our study aimed to understand the role of *Foxg1* in OL development and myelination during the early postnatal period. We found that deficiency of *Foxg1* initially led to reduced myelination and a lower number of mature OLs, but more OLIG2^+^ total OL lineage cells and PDGFRα^+^ OPCs. These effects were transient and primarily occurred in the first two weeks after birth. Additionally, we identified potential molecular pathways involved in these effects, including impaired PDGFRα signaling attenuation, reduced OPC cell cycle exit, and altered expression of Hes5, Olig2, and Sox10. Our study demonstrates the significance of *Foxg1* in regulating the timely onset of myelination and provides new insights into the pathogenesis and potential therapeutic strategies for *FOXG1* syndrome.

### 3.1. Exploring OL Dysfunction in FOXG1 Syndrome from Animal Models

Despite the abundance of published evidence supporting delayed myelination in *FOXG1* syndrome, the majority of these findings are derived from neuroimaging studies of *FOXG1* syndrome patients [27,28]. It is challenging to gain a comprehensive understanding of OL dysfunction during disease progression. Therefore, the establishment of an animal model that can simulate the neuropathologic symptoms of *FOXG1* syndrome is essential for exploring the underlying mechanisms and developing effective therapeutic strategies. Recently, Wilpert et al. [29] provided the first comprehensive report of postmortem neuropathologic findings in two fetuses with *FOXG1* syndrome. They observed an increase in PDGFRα^+^ OPCs and a decrease in pre-OLs in two *FOXG1*-haploinsufficient patients, indicating an abnormal OL maturation process. Our findings in *Foxg1* cKO mice, which show a decrease in CC1^+^ terminally differentiated OLs and an increase in OPCs, are consistent with their findings. Our experimental model is valuable for investigating the molecular and cellular mechanisms that contribute to the compromised development and functioning of OLs in *FOXG1* syndrome.

We found that *Foxg1* cKO mice exhibited reduced myelination and increased OPC numbers in several brain regions, including the CC and cortex (motor and cingulate cortex), during the first two weeks after birth. This may affect communication with other brain regions and account for some abnormal behavior observed in *FOXG1* syndrome, including limb spasms, irritability, and language impairments [30,31,32,33]. *FOXG1* syndrome is a neurodevelopmental disorder that causes severe autism spectrum disorder (ASD)-like symptoms. Many studies have found that in ASD, OPCs are normally generated but fail to mature properly into functional myelinating cells, resulting in aberrant myelination [34,35,36]. Our study also supports these findings and suggests that impaired OPC maturation contributes to abnormal myelination in *FOXG1* syndrome.

The present study reveals the crucial cell-autonomous role of *Foxg1* in myelination in mice. We use a novel *NestinCreER;Foxg1^fl/fl^;Sox10^GFP/tdTOM^* triple-transgenic mouse model, which allows us to selectively delete *Foxg1* in NPCs and track their fate in the OL lineage. In this way, we could compare the morphology and MBP expression of OLs derived from *Foxg1*-deleted and non-deleted NPCs in combination with immunostaining. The limitation of this mouse model is that it requires the integration of three transgenic components, which may pose some challenges and variability in gene expression. However, this does not affect the validity and significance of our findings. In the future, based on the tracer role of RFP and GFP, we can use this triple-labeled transgenic mouse model to observe the dynamic changes of both *Foxg1*-deleted and non-deleted OLs, as well as their interactions with neurons, under in vivo or in vitro conditions, using fluorescence microscopy. Additionally, this mouse model can be used to investigate the effects of *Foxg1* on the electrophysiological properties of OLs. However, *FOXG1* syndrome results from global functional abnormalities or deficiencies of *FOXG1*, which can affect different cell types, including neurons, astrocytes, and even possibly microglia, and occurs mostly de novo during early embryogenesis [37]. To better understand the role of *FOXG1* in myelination, it is necessary to establish animal models that are more relevant to *FOXG1* syndrome. On the other hand, FOXG1 syndrome is a rare neurodevelopmental disease, and understanding its progression and mechanisms solely based on imaging studies and limited clinical cases poses challenges. More comprehensive clinical data will be needed in the future from larger patient cohorts to corroborate and contextualize experimental findings.

### 3.2. Foxg1 Modulates OL Diversely in Myelination and Remyelination

A recent study conducted by Dong et al. [19] has revealed the significant regulatory role of *Foxg1* in the differentiation and maturation of OPCs during the processes of demyelination and remyelination. In their work, the deletion of *Foxg1* not only reduced CPZ-induced demyelination but also accelerated the remyelination process in adult mice. The researchers observed that *Foxg1* deletion led to a decrease in OPC proliferation and an enhancement of their differentiation into mature OLs, both in vivo and in vitro. The importance of *Foxg1* in the differentiation and maturation of OLs and myelination has been a subject of great interest. Interestingly, its effects seem to vary depending on different developmental stages or pathological conditions. Additionally, we cannot completely rule out the possibility that these mice in our study may show increased myelination at a later stage. In our study, we followed the myelination process until P30, and we speculate that myelin may continue to increase beyond the observed time point as more OPCs differentiate into mature OLs and produce myelin sheaths. Similar observations have been reported in other gene knockout mouse models, such as the *Fmr1* knockout mice and the fragile X syndrome mouse model [38]. Despite an initial delay in the first two weeks after birth, the myelination level of the *Fmr1* cKO mice catches up to that of wild-type mice at one month of age. Interestingly, at 2–4 months of age, the myelin protein level of the *Fmr1* cKO mice significantly exceeds that of wild-type mice. We speculate that this may be related to the delayed maturation of OPCs.

Dong et al. [19] propose that *Foxg1* promotes the repair of demyelinating injury by upregulating OL differentiation, which is mediated by components of the Wnt signaling pathway, including GSK3β and β-catenin. Our study did not find any alterations in the levels of GSK3β and β-catenin. We also speculated that *Foxg1* might influence OL differentiation by modulating other factors of the Wnt signaling pathway, such as Wnt3a and Tcf4, but no significant alteration in their expression levels was observed. One possibility to consider is that different mechanisms of myelination may contribute to these variations. For example, developmental myelination is a natural process during CNS development and depends on the proliferation, migration, and maturation of newly generated OPCs, whereas remyelination occurs as a response to demyelination and depends mainly on the activation and differentiation of existing OPCs [5,39,40]. *Foxg1* may play different roles in these two processes by regulating different signaling pathways and transcription factors. A similar phenomenon of conditional deletion of *Foxg1* playing opposite roles in myelination and remyelination was seen in mice with conditional knockout of p38, a mitogen-activated protein kinase, suggesting a possible partial similarity in their regulatory mechanisms in these processes [41].

### 3.3. Potential Mechanisms of Foxg1 Regulation on Developmental Myelination

Our study shows that the loss of *Foxg1* results in the failure of timely downregulation of PDGFRα expression and a significant reduction in cell cycle exit. PDGFRα is highly expressed in OPCs and activates its tyrosine kinase activity by binding to members of the PDGF family, thereby maintaining OPCs proliferation and survival [42,43]. Additionally, PDGFRα physically interacts with proteoglycan NG2 to enhance the activation of the PDGF signaling pathway [44]. As OPCs undergo terminal differentiation and exit the cell cycle, PDGFRα expression rapidly decreases [45]. Understanding the mechanisms underlying the downregulation of PDGFRα expression is crucial for OPC differentiation. Several transcription factors and enzymes are involved in downregulating PDGFRα expression [46,47]. For example, Nkx2.2 is a homeodomain transcription factor that binds directly to the PDGFRα promoter, inhibiting its gene transcription. How *Foxg1* regulates PDGFRα expression, whether via direct transcriptional repression or synergistic interaction with other factors such as Nkx2.2, requires further investigation.

Previous studies have shown that increased expression of PDGFRα can lead to insufficient myelin sheath generation, potentially due to the inhibition of myelin-promoting factors such as Olig2 and Sox10 [48]. However, in our study, we observed an unexpected increase in the expression of Olig2 and Sox10 despite the increased expression of PDGFRα. A possible explanation for this discrepancy is that *Foxg1* deficiency activates differential transcriptomic regulation networks that modulate the expression of Olig2, Sox10, and PDGFRα. FOXG1 is known to act as a transcriptional repressor for various proteins [13,49]. For instance, in mouse neural stem cells, FOXG1 directly binds to the promoters of crucial transcription factors such as SOX9, ZBTB20, and NFIA, inhibiting their expression and determining the fate of stem cells toward the astroglial lineage [22]. Therefore, we speculate that FOXG1 may have a similar repressive role on OLIG2 and SOX10 and that the loss of FOXG1 relieves the repression of Olig2 and Sox10.

Regarding inhibitory factors, we observed not only significantly upregulated PDGFRα but also moderately increased Hes5. Hes5 acts as an effector molecule in the Notch signaling pathway, which inhibits OL differentiation and myelination by repressing the expression of myelin genes and interacting with other transcription factors, such as Mash1 and Sox10 [23]. Similar to the role of PDGFRα in OPCs, Hes5 expression decreases during OPC differentiation, and its overexpression can prevent this process [50]. Previous research has indicated that the Notch signaling pathway plays a crucial role in various stages of generating mature, myelinating OLs [51,52]. The involvement of the Notch signaling pathway in the delayed OL development that results from *Foxg1* deficiency is an interesting avenue to explore.

We proposed that the upregulation of Olig2 and Sox10 after *Foxg1* deletion induced the differentiation of a subset of NPCs into OPCs. This, along with the effects of Pdgfrα and hes5 on OPC proliferation and survival, may account for the observed increase in the population of PDGFRα^+^ OPCs and OLIG2^+^ cells. We found that *Foxg1* deletion had a dual role in OL development: it facilitated the transition of NPCs to OPCs but inhibited the exit of OPCs from the cell cycle. These findings suggest that *Foxg1* regulates OL development in a stage-specific manner. However, these results are preliminary as *Foxg1* is implicated in various cellular processes, and its effects can vary depending on developmental stages, cell types, and pathological conditions. Fully mapping its diverse molecular interactions and cellular outputs remains an important and challenging goal for future studies. To gain more insights into the role of *Foxg1* in OL development and myelination, future studies can employ more sophisticated and comprehensive techniques to explore novel targets. For example, transcriptomic and epigenetic analyses can be performed to identify the global gene expression and chromatin changes induced using *Foxg1* deletion in OL lineage cells. Moreover, specific transgenic tools can be used to manipulate *Foxg1* expression in a temporally and cell-type-specific manner.

It should be noted that there are still certain limitations in our study to be considered. While providing initial evidence for the role of *Foxg1* in oligodendrocyte development and myelination, our study only examined the effects of *Foxg1* deficiency in mice up to P30. Further long-term studies beyond this early developmental window will be important for fully understanding the consequences of *Foxg1* deficiency on myelin integrity and disease progression. In addition, *FOXG1* syndrome is a clinically heterogeneous disorder, and the diverse symptoms may arise not only from *Foxg1* and OLs dysfunction but also from additional genetic, epigenetic, and environmental factors influencing neurodevelopment, suggesting that the underlying etiology of *FOXG1* syndrome is likely multifactorial. Further research is needed to fully elucidate the complex pathophysiology underlying this disorder.

In conclusion, our study provides new insights into the role of *Foxg1* in myelin development and uncovers potential pathological mechanisms of *FOXG1* syndrome, which have important implications for the development of therapeutic strategies not only for *FOXG1* syndrome but also for other neurodevelopmental disorders associated with abnormal myelination. 

## 4. Materials and Methods

### 4.1. Animals

Conditional deletion of *Foxg1* was achieved by crossing *Nestin-creER* (Stock No. 016261) mice with *Foxg1^fl/fl^* mice [53] and administering tamoxifen using intraperitoneal injection (Sigma–Aldrich, T5648-5G, Louis, MO, USA) at a dose of 5 μL/g body weight on the day of birth (designated as P0) using a 20 mg/mL solution prepared in corn oil (Sigma–Aldrich, C8267). To track *Foxg1*-deficient cells, the *ROSA26-YFP* reporter line (Stock No. 006148) was introduced. The *NestinCreER;Foxg1^fl/fl^* mice, and the *Foxg1^fl/fl^* mice were referred to as *Foxg1* cKO and control mice, respectively. The triple-transgenic *Nestin-CreER;Foxg1^fl/fl^;Sox10^+^/^GFP/tdTom^* mice were obtained by crossing *Foxg1^fl/fl^* and *Sox10^GFP/tdTom^* mice [54] to generate F1 mice carrying both *Foxg1^fl/+^* and *Sox10^GFP/tdTom^* alleles. Then, F1 mice were crossed with *Nestin-creER;Foxg1^fl/+^* mice to obtain F2 mice carrying *Nestin-creER;Foxg1^fl/fl^* and *Sox10^GFP/tdTom^* alleles.

### 4.2. Immunofluorescence Staining

Mouse brain tissues were fixed in 4% paraformaldehyde overnight at 4 °C and then cryoprotected in 30% sucrose solution for 48 h at 4 °C. The tissues were embedded in OCT compound (Sakura) in a square aluminum box, rapidly frozen in liquid nitrogen, and stored in a −80 °C freezer until ready for sectioning. The tissues were sectioned at a thickness of 20 μm using a Leica CM 3050S cryostat. The sections were blocked with 5% normal goat serum in PBS containing 0.1% Triton X-100 for 1 h at room temperature. Primary antibodies were incubated with the sections overnight at 4 °C. The primary antibodies used were as follows: rabbit anti-FOXG1 (Abcam, 1:500, AB18259, Cambridge, UK), mouse anti-OLIG2 (Millipore, 1:200, MABN50, Billerica, MA, USA), rat anti-MBP (Abcam, 1:1000, AB7349), rabbit anti-KI67 (Abcam, 1:500, AB16667), rat anti-BrdU (Abcam, 1:1000, AB6326, Cambridge, UK), mouse anti-SOX2 (Santa Cruz, 1:200, sc-365823, Dallas, TX, USA), mouse anti-CC1 (GeneTex, 1:500, GTX16794, Irvine, CA, USA), mouse anti-PDGFRα (BD Pharmingen, 1:500, 558774, San Diego, CA, USA), rabbit anti-RFP (Abcam, 1:500, AB62341), chicken anti-GFP (Abcam, 1:1000, AB13970). After washing with PBS, the sections were incubated with Alexa Fluor-conjugated secondary antibodies for 2 h at room temperature. The secondary antibodies used were: Alexa Fluor 555-conjugated donkey anti-mouse (Invitrogen, 1:500, A32773, Waltham, MA, USA), Alexa Fluor 647-conjugated donkey anti-mouse (Invitrogen, 1:500, A32787), Alexa Fluor 555-conjugated donkey anti-rat (Invitrogen, 1:500, A48270), Alexa Fluor 488-conjugated donkey anti-rat (Invitrogen, 1:500, A48269), Alexa Fluor 488-conjugated goat anti-chicken (Invitrogen, 1:500, A11039), Alexa Fluor 555-conjugated donkey anti-goat (Invitrogen, 1:500, A21432), Alexa Fluor 647-conjugated donkey anti-rabbit (Invitrogen, 1:500, A31573), Alexa Fluor 555-conjugated donkey anti-rabbit (Invitrogen, 1:500, A31572). The nuclei were counterstained with DAPI (Sigma–Aldrich, 10236276001, Louis, MO, USA). The images were acquired using an Olympus FV1000 confocal microscope (Tokyo, Japan) and an Olympus VS200 digital slide scanner (Tokyo, Japan). Image analysis and processing were performed using Image J software 1.52a (US National Institutes of Health, Bethesda, MD, USA).

### 4.3. BrdU Administration

BrdU (Sigma–Aldrich, B5002) was dissolved in saline at a concentration of 10 mg/mL. We performed BrdU (50 mg/kg) labeling of S-phase OPCs using intraperitoneal injection 36 h prior to analysis in P8 mice.

### 4.4. Quantitative Real-Time PCR

Total RNA was extracted from mouse forebrain tissues at P8 using the RNeasy Plus Mini Kit (Qiagen, 74104, Hilden, Germany). The RNA concentration and quality were measured using a NanoDrop spectrophotometer (Thermo Fisher Scientific, Waltham, MA, USA). One microgram of total RNA was reverse transcribed to cDNA using the PrimeScript RT Reagent Kit with gDNA Eraser (Takara, RR047A, Kusatsu, Japan). Real-time qPCR was performed on a StepOne Real-Time PCR System (Applied Biosystems, Foster City, CA, USA) in accordance with the manufacturer’s instructions. The reaction mixture comprised Roche’s qPCR Master Mix (Roche, 4913850001, Basel, Switzerland) and specific primers designed for the target genes. The primer sequences were listed in the Supporting Information Table S1. The relative expression levels of target genes were normalized to the expression of GAPDH and calculated using the 2^−ΔΔCt^ method.

### 4.5. Western Blotting

Mouse forebrain tissues were homogenized in RIPA buffer (Beyotime, P0013B, Shanghai, China) containing protease and phosphatase inhibitors (Roche, SKU49068, Basel, Switzerland). Protein concentration was determined using BCA assay (Thermo Fisher Scientific, 23225, Waltham, MA, USA). Equal amounts of protein (20 μg) were separated using SDS-PAGE and transferred to PVDF membranes (Millipore). The membranes were blocked with 5% non-fat milk in TBST for 1 h at room temperature and then incubated with primary antibodies overnight at 4 °C. The primary antibodies used were as follows: rat anti-MBP (Abcam, 1:5000, AB7349) and rabbit anti-β-tubulin (Cell Signaling Technology, 1:5000, 2146s). After washing with TBST, the membranes were incubated with HRP-conjugated secondary antibodies: goat anti-rat (Abcam, 1:10000, AB205720) or goat anti-rabbit (Cell Signaling Technology, 1:5000, 7074, Danvers, MA, USA) for 1 h at room temperature. Signals were detected using an enhanced chemiluminescence (ECL) kit (Thermo Fisher Scientific, 32106) and quantified using ImageJ software 1.52a.

### 4.6. In Situ Hybridization

In situ hybridization was performed on mouse forebrain sections using traditional methods. Briefly, mouse brain tissue was fixed in paraformaldehyde, cryoprotected in sucrose solution, embedded in OCT compound, and sectioned at 20 μm thickness using a Leica CM 3050S cryostat. Sections were incubated with proteinase K (10 μg/mL) for 30 min at 37 °C. Prehybridization was performed at 65 °C for 2 h in a hybridization buffer. The sections were then hybridized overnight at 65 °C with digoxigenin-labeled RNA probes specific to the target gene. After hybridization, the sections were washed three times in 2× SSC at 65 °C for 30 min each, followed by blocking with 5% sheep serum and incubation with anti-digoxigenin-AP antibody (Roche, 1:5000, 11333089001) overnight at 4 °C. Signal detection was performed using the NBT/BCIP substrate. The plasmid used to synthesize the probes of mouse Mbp, Plp1, and Pdgfrα in this study was generously provided by Dr. Mengsheng Qiu from Hangzhou Normal University [55]. Images were acquired using a bright field microscope (Olympus BX53, Japan) and a digital slide scanner (Olympus VS200, Japan).

### 4.7. Cell Count and IOD Analysis

For each experiment, three to four pairs of brains from different litters were utilized. To ensure that our results were representative of the entire region of interest, we analyzed multiple slices from each brain region. A defined area was analyzed using ImageJ software 1.52a in three coronal sections selected from each brain between bregma 1.10 and −0.10 mm. For the analysis of MBP integral optical density (IOD) and cell cycle exit index analysis at P8, we focused on the external capsule, which is an area in the forebrain where OPCs actively proliferate and initiate the myelination process. In the immunofluorescence staining and in situ hybridization experiments at P15 and P30, the CC and cortex (including motor and cingulate cortex) were selected as regions of interest for counting oligodendrocyte lineage cells (OLIG2^+^, CC1^+^, PLP1^+^, and PDGFRα^+^ cells). The cell counting was performed using ImageJ software 1.52a. In order to minimize errors resulting from parameter adjustments during the counting process, two independent researchers, who were blinded to the experimental conditions, conducted the cell counting for each sample. 

### 4.8. Statistical Analysis

All data were analyzed using GraphPad Prism version 8.0. The normality of data distribution was validated using the Shapiro–Wilk test. Statistical comparisons between two independent groups were performed using unpaired two-tailed Student’s *t*-test. For comparisons among multiple groups, a one-way analysis of variance followed by Bonferroni’s multiple comparisons post hoc test was used. All quantitative data are expressed as mean ± standard error of the mean. 

## Data Availability

Data are available from the corresponding author on request.

## Supplementary Materials

The supporting information can be downloaded at: https://www.mdpi.com/article/10.3390/ijms241813921/s1.
