# Supplementary material for: Conditional Deletion of Foxg1 Delayed Myelination during Early Postnatal Brain Development
Source: Int J Mol Sci. 2023 Sep 10;24(18):13921. doi: 10.3390/ijms241813921 (PMC10530892; doi:10.3390/ijms241813921)
Supplement: Supplementary file 1 [file ijms-24-13921-s001.zip › Table S1 Primers used for real-time qPCR.pdf]

Table S1 Primers used for real-time qPCR

Gene	Forward	Reverse
GAPDH	AGGTCGGTGTGAACGGATTTG	TGTAGACCATGTAGTTGAGGTCA
Notch1	GCAACTGTCCTCTGCCATATAC	GTCTTCAGACTCCTTGCATACC
Hes1	GAGGCGAAGGGCAAGAATAA	GAATGCCGGGAGCTATCTTT
Hes5	AGTCCCAAGGAGAAAAACCGA	GCTGTGTTTCAGGTAGCTGAC
Id2	CCCGGTGGACGACCCGATG	CAGATGCCTGCAAGGACAGGATGC
Id4	CAGTGCGATATGAACGACTGC	GACTTTCTTGTTGGGCGGGAT
Gsk-3β	TGGCAGCAAGGTAACCACAG	CGGTTCTTAAATCGCTTGTCCTG
β-catenin	ATGGAGCCGGACAGAAAAGC	CTTGCCACTCAGGGAAGGA
Tcf4	AACGATGATGAGGACCTGAC	CAGCTTTCGGGTTCAGATTC
Sox10	ATAACCTCATCCCTTGCCTAAC	TGGATTGCCTCTGACTCTTTC
Pdgfra	AGAGTTACACGTTTGAGCTGTC	GTCCCTCCACGGTACTCCT
Olig2	GAAGCAGATGACTGAGCCCGAG	CCCGTAGATCTCGCTCACCAG
Myrf	CCTGTGTCCGTGGTACTGTG	TCACACAGGCGGTAGAAGTG
Sox4	CGGCTGCATCGTTCTCTCC	GGTAGACGTGCTTCACTTTCTTG
Wnt3a	CTCCTCTCGGATACCTCTTAGTG	GCATGATCTCCACGTAGTTCCTG
